# Phenotype Selection Reveals Coevolution of Muscle Glycogen and Protein and PTEN as a Gate Keeper for the Accretion of Muscle Mass in Adult Female Mice

**DOI:** 10.1371/journal.pone.0039711

**Published:** 2012-06-29

**Authors:** Mandy Sawitzky, Anja Zeissler, Martina Langhammer, Maximilian Bielohuby, Peggy Stock, Harald M. Hammon, Solvig Görs, Cornelia C. Metges, Barbara J. M. Stoehr, Martin Bidlingmaier, Carolin Fromm-Dornieden, Bernhard G. Baumgartner, Bruno Christ, Bertram Brenig, Gerhard Binder, Friedrich Metzger, Ulla Renne, Andreas Hoeflich

**Affiliations:** 1 Laboratory for Mouse Genetics, Research Unit Genetics & Biometry, Leibniz Institute for Farm Animal Biology (FBN), Dummerstorf, Germany; 2 Endocrine Research Unit, Medizinische Klinik und Poliklinik IV, Klinikum der Ludwig-Maximilians University, Munich, Germany; 3 ZAMED, Molecular Hepatology, Martin-Luther University, Halle, Germany; 4 Research Unit Nutritional Physiology, Leibniz Institute for Farm Animal Biology (FBN), Dummerstorf, Germany; 5 Molecular Biology, Institute of Veterinary Medicine, Göttingen, Germany; 6 Pediatric Endocrinology, University-Children's Hospital, Tübingen, Germany; 7 F. Hoffmann-La Roche Ltd., CNS Discovery Research, Basel, Switzerland; Rutgers University, United States of America

## Abstract

We have investigated molecular mechanisms for muscle mass accretion in a non-inbred mouse model (DU6P mice) characterized by extreme muscle mass. This extreme muscle mass was developed during 138 generations of phenotype selection for high protein content. Due to the repeated trait selection a complex setting of different mechanisms was expected to be enriched during the selection experiment. In muscle from 29-week female DU6P mice we have identified robust increases of protein kinase B activation (AKT, Ser-473, up to 2-fold) if compared to 11- and 54-week DU6P mice or controls. While a number of accepted effectors of AKT activation, including IGF-I, IGF-II, insulin/IGF-receptor, myostatin or integrin-linked kinase (ILK), were not correlated with this increase, phosphatase and tensin homologue deleted on chromosome 10 (PTEN) was down-regulated in 29-week female DU6P mice. In addition, higher levels of PTEN phosphorylation were found identifying a second mechanism of PTEN inhibition. Inhibition of PTEN and activation of AKT correlated with specific activation of p70S6 kinase and ribosomal protein S6, reduced phosphorylation of eukaryotic initiation factor 2α (eIF2α) and higher rates of protein synthesis in 29-week female DU6P mice. On the other hand, AKT activation also translated into specific inactivation of glycogen synthase kinase 3ß (GSK3ß) and an increase of muscular glycogen. In muscles from 29-week female DU6P mice a significant increase of protein/DNA was identified, which was not due to a reduction of protein breakdown or to specific increases of translation initiation. Instead our data support the conclusion that a higher rate of protein translation is contributing to the higher muscle mass in mid-aged female DU6P mice. Our results further reveal coevolution of high protein and high glycogen content during the selection experiment and identify PTEN as gate keeper for muscle mass in mid-aged female DU6P mice.

## Introduction

### Critical Role of AKT for Muscle Accretion

AKT represents the key factor of muscle accretion and maintenance [1], [2]. AKT 1 and AKT 2 double knockout mice were characterized by severe growth retardation, reduced muscle mass, particularly due to smaller muscle fiber volumes and perinatal lethality [3]. On the other hand, overexpression of AKT in transgenic mice resulted in muscle fiber hypertrophy [4] and higher muscle strength [5]. Interestingly, knockdown of AKT 1 in cell culture studies completely prevented myoblast differentiation but had no effect on cell proliferation and it was concluded that AKT 1 is particularly relevant for muscle formation in embryonic development [6], [7].

### Control of AKT

The activity of AKT 1 is controlled by phosphorylation of threonine residue 308 and serine residue 473 [8]. While Thr-308 is phosphorylated by phosphoinositide dependent kinase 1 (PDK1) as part of the PI3 pathway [9], Ser-473 can be activated by different kinases. It has been demonstrated, that mammalian target of rapamycin (mTOR) in complex 2 (TORC2; [10]) or integrin-linked kinase (ILK) [11] can phosphorylate Ser-473 present in AKT 1. Furthermore, it was suggested that TGFß1 affects Ser-473 phosphorylation of AKT 1 via upregulation of ILK [12]. TGFß has recently also been shown to block gene expression of IGF-II in myoblasts [13] suggesting different levels of interaction for TGFß and muscle differentiation. Interestingly, within the cell activation of Ser-473 AKT has inversely been correlated with expression of PTEN [14]. PTEN, which primarily is recognized as a tumor suppressor, acts by dephosphorylating PIP3 and thereby prevents activation of PDK1 on the one hand and recruitment of AKT to the plasma membrane on the other [15].

Prominent functions for muscle growth and control of AKT have been attributed particularly to IGF-I, IGF-II and to growth differentiation factor-8 (myostatin). The latter is mutated in extreme muscle phenotypes as found in Belgian Blue or Piedmontese cattles [16].

### Effects of AKT

Activated AKT affects protein metabolism via at least two independent mechanisms: First, AKT can activate ribosomal protein S6 via mTOR and p70 ribosomal protein S6 kinase (p70S6K). Secondly, AKT also affects protein translation through phosphorylation of GSK3ß [17], [18]. AKT also exerts affects on glucose metabolism and controls GLUT4 dependent glucose uptake [19], [20] and glycogen synthesis via GSK3ß and glycogen synthase [21], [22].

### Models to Study Control of Protein Accretion

Functional genome analysis by the employment of a number of loss or gain of function models *in vitro* and *in vivo* have attributed specific functions of isolated growth factors or receptors for the accretion and maintenance of muscle mass [23], [24]. However the limitations of such reverse genetics are due to the fact, that isolated gene effects do not merge genetic complexity present in real life. In addition, transgenic or knockout models do not contain reliable information on the physiological relevance of a derived hypothesis. Thus we used a mouse model established by means of classical genetics [25], [26] and asked, which from the different mechanisms show up with the phenotype of high protein mass. Since the establishment of our model was originally based on an outbred genetic background, we presume the presence of multiple mechanisms attributing to the robust phenotype. In spite of 138 generations of phenotype selection we identified the presence of one specific mechanism potentially affecting activity of AKT arguing against outmost biodiversity as a mechanism of higher protein accretion in our system. In addition, our experiment argues in favor of an intricate relationship between protein and glycogen metabolism in muscle tissue.

## Materials and Methods

### Animals

All procedures were done in accordance to national and international guidelines and approved by our own institutional board (Animal Protection Board from the Leibniz-Institute for Farm Animal Biology) and by the national Animal Protection Board Mecklenburg-Vorpommern (file number: LALLF M-V/TSD/7221.3-1.2-037/06). We used a non inbred mouse line (DU6P) established by phenotype selection over 138 generations for high protein mass at an age of 42 d after birth [27], [28]. In addition, an unselected control mouse line Fzt:DU (generation 142) was used. The initial population was derived from an original crossbred of four outbred (NMRI orig., Han:NMRI, CFW, CF1) and four inbred (CBA/Bln, AB/Bln, C57BL/Bln, XVII/Bln) populations. In brief, during the whole selection experiment for every generation 60–80 breeds were established. In order to maintain maximal genetic complexity all breeds had small inbred coefficients as calculated by an in house computer program. From every litter two male mice were randomly selected and analyzed for the total protein amount of the carcass (without skin, head and all inner organs). Only littermates from mice characterized by the highest protein amounts were selected for the next round of selection. The selection intensity as a function of litter quality varied between 50% and 70%. The mice were maintained in a semi-barrier system and had free access to a standard diet (breeding diet 1314, Altromin, Lage, Germany) and water *ad libitum*.

### Longitudinal Study Design and Sampling Regime

A longitudinal study was performed by dissecting male and female mice (n = 15) at 8 time points (2, 4, 7, 11, 16, 29, 42, 54 weeks). The body weights were documented in monthly intervals and at dissection. In addition, at dissection weights of carcass and *Musculus quadriceps femoris* were recorded, the latter was stored at −80°C until analysis. The carcass represents the body with muscles and bones after removal of the skin and inner organs.

### Western Immunoblot Analysis

*Musculus quadriceps femoris* was homogenized in lysis buffer (#9803, New England Biolabs, Frankfurt, Germany) supplemented with protease inhibitor cocktail (Complete Mini, Roche, Mannheim, Germany) using the Precellys24 (Peqlab Biotechnologie GmbH, Erlangen, Germany). After centrifugation (10000 g, 2 min, 4°C) and elimination of cell debris, protein content of the whole tissue lysate was quantified using the bicinchoninic acid method as described previously [29].

Twenty micrograms of protein from the pool samples were separated on 12% SDS-PAGE gels and transferred to polyvinylidene fluoride membranes (Millipore, Eschborn, Germany). Equal loading of the gels and proper transfer of the proteins to the membranes were verified by Coomassie Blue staining. Membranes were blocked in 5% dry milk and 1% Tween20 dissolved in Tris buffered saline/TBST. After three washings in TBST buffer the membranes were incubated with primary antibodies for 1.5 hours at room temperature. We analyzed protein expression and phosphorylation by using a set of different antibodies (see table 1). All antibodies were purchased from Cell Signaling Technology (New England Biolabs, Frankfurt, or St. Cruz, Heidelberg, Germany) and have been used according to the manufacturer’s instructions.

**Table 1:** Antibodies used in the study.

Phospho-specific antibodies	Source	Order number #	Company
AKT-Ser473	rabbit	9271	Cell Signaling
AKT-Thr308	rabbit	9275	Cell Signaling
GSK3ß-Ser9	rabbit	9336	Cell Signaling
PTEN-Ser380	rabbit	9551	Cell Signaling
p44/42 MAPK-Thr202/204	rabbit	4370	Cell Signaling
p38 MAPK-Thr180/182	rabbit	9211 and 4511	Cell Signaling
PDK1-Ser241	rabbit	3061	Cell Signaling
eIF2α-Ser51	rabbit	3398	Cell Signaling
IGF-IRß/Insulin-Rß-Tyr1135/1136-1150/1151	rabbit	3024	Cell Signaling
S6 Ribosomal Protein-Ser235/236	rabbit	4858	Cell Signaling
p70S6 Kinase-Thr389	rabbit	9234	Cell Signaling
**Phospho-unspecific antibodies**	**Source**	**Order number #**	**Company**
AKT	rabbit	9272	Cell Signaling
GSK3ß	rabbit	9315	Cell Signaling
PTEN	rabbit	9552 and 9188	Cell Signaling
p44/42 MAPK	rabbit	4695	Cell Signaling
p38 MAPK	rabbit	9212	Cell Signaling
PDK1	rabbit	3062	Cell Signaling
eIF2α	mouse	2103	Cell Signaling
IGF-IRß	rabbit	3018	Cell Signaling
S6 Ribosomal Protein	rabbit	2217	Cell Signaling
p70S6 Kinase	rabbit	2708	Cell Signaling
ILK	rabbit	3856	Cell Signaling
α-Tubulin	rabbit	2125	Cell Signaling
Ubiquitin	mouse	3963	Cell Signaling
GDF-8	rabbit	sc-6885	Santa Cruz
IGF-II	mouse	sc-74119	Santa Cruz

After three washings in TBST the membranes were incubated with horseradish peroxidase coupled goat anti-rabbit IgG (1∶2000, #7074) or horse anti-mouse IgG (1∶5000, #7076) for 1 h at room temperature. Finally, membranes were developed on a Kodak Image Station 4000 MM (Stuttgart, Germany) using an enhanced chemiluminescence detection kit (ECL Advance Western Blotting Detection Kit; GE Healthcare, Freiburg, Germany). Band intensities were quantified using the ImageQuant Software package (GE Healthcare). To estimate the specific activation for distinct signaling molecules, signals from phospho-specific antibodies were normalized for total protein expression (phospho/total protein, see table 1). Therefore, membranes were initially incubated with phospho-specific antibodies. The same membrane was then reprobed by using antibodies directed against the total protein. Results are presented in percent of phospho/total protein or total protein/loading control.

### Isolation of the Membrane Fraction

Plasma membranes were prepared as described previously [30]. Muscle tissue were homogenized 2 times for 54 sec in TRIS buffer (10 mM, pH 7.4) containing protease inhibitor cocktail (Complete Mini, Roche, Mannheim, Germany) using the gentleMACS™ dissociator system (Mylteni Biotec GmbH, Bergisch-Gladbach, Germany). Cell debris and nuclei were separated by centrifugation (10000 g, 10 min, 4°C). The supernatant containing plasma membranes and the cytosolic fraction was separated in a final centrifugation step for 30 min at 17530 g and 4°C. The efficient removal of cytosolic proteins in isolated plasma membranes was demonstrated by the absence of cytosolic GAPDH (data not shown).

### Analysis of Muscle Glycogen

Glycogen content was quantified in muscle samples of 29-week female Fzt:DU (n = 6) and DU6P mice (n = 12) using the Starch kit (#10207748035, charge number 11849700, Boehringer Mannheim/R-Biopharm, Germany) according to the manufacturers instructions. Furthermore, muscle glycogen was also visualized by histochemistry. Serial transverse sections (5 µm) were cut from muscle samples of 29-week female Fzt:DU and DU6P mice with a MEV cryostat (SLEE Technical GmbH, Mainz, Germany) at −20°C and mounted on slides. Glycogen was detected by performing the Perdiodic Acid Schiff (PAS) staining. Sections were affixed by 5 min incubation in methanol/acetone 1∶1 at −20°C and washed for 5 min with double distilled water. The slides were incubated for 5 min with freshly prepared 1% periodic acid (Carl Roth GmbH + Co. KG, Karlsruhe, Germany). After a 5 min washing step with aqua dest., the slides were incubated in Schiff reagent (Merck, Darmstadt, Germany) for 10 min. The slides were then washed in tap water (∼35°C), counterstained with Mayer’s hematoxylin (Merck, Darmstadt, Germany) for 30 sec and washed again with tap water for 5 min. Finally, the slides were mounted with Aquatex (Merck, Darmstadt, Germany) and muscle glycogen was visualized by light microscopy (Nikon, Eclipse E600, Lucia G software).

### Measurement of Serum IGF-I and IGF-II

Total IGF-I in serum of female mice (n = 8) was measured in triplicates with a commercially available ELISA as per manufacturer’s instructions (mouse IGF-I assay #E25, Mediagnost, Reutlingen, Germany). Circulating IGF-II concentrations in Fzt:DU and DU6P mice (n = 10) have been measured by RIA as described before [31].

### Quantification of Muscle RNA and DNA Contents

To isolate RNA of *Musculus quadriceps femoris* (n = 5), a defined amount of pestled tissue was homogenized with TRIreagent (Sigma, Hamburg, Germany) and incubated for 5 min at room temperature (RT). After centrifugation for 10 min at 12000 g supernatants were vortexed in 100 µl Chloroform, incubated for further 10 min at RT and centrifuged (12000 g, 10 min). The isolated liquid phase was mixed with 500 µl isopropanol and after 5 min (RT) another centrifugation step at 12000 g for 8 min was performed. The achieved pellet was washed with 75% ethanol, centrifuged (7500 g, 5 min), air-dried and resolved in 50 µl sterile water.

DNA of a defined amount of *Musculus quadriceps femoris* was isolated with the High Pure PCR Template Preparation Kit according to manufacturer’s instructions (Roche, Mannheim, Germany). RNA and DNA contents were analysed by using the Nanodrop ND-1000 spectrophotometer (Peqlab Biotechnologie GmbH, Erlangen, Germany).

### Analysis of Translational Activity

Flash frozen mouse muscle tissue was lysed mechanically in polysome buffer (300 mM KCl, 5 mM MgCl_2_, 10 mM PIPES pH 7.4), 0.5% NP40, 27.8 mg/ml Heparin-Sodium (Ratiopharm, Ulm, Germany) and 100 ng/ml Cycloheximide (Sigma, Munich, Germany) with Tissue Lyser LT (Qiagen, Hilden, Germany). To separate polysomal RNA from non-polysomal RNA, linear sucrose gradients were built from polysome buffer with 0% to 50% sucrose concentration [32]. Stability of linear gradients was confirmed by a refractometer (Type MHRB 90, Müller, Erfurt, Germany). Muscle lysate was cooled on ice and layered onto gradients which were subjected to centrifugation at 28000 g in a SW40 rotor (Beckmann Optima™ L Preparative Ultracentrifuge, Krefeld, Germany) at 4°C for 120 min. 13 fractions of 1 ml were collected from the top of the gradient and the ratio of 18S and 28S rRNA was measured to obtain the polysome profile on Agilent 2100 Bioanalyzer (Waldbronn, Germany). Based on the 28S/18S Ratio, fractions 5 to 6 (from top of gradient) were described as non-polysomal (ratio ≠ 2) and fractions 9 to 11 as polysomal (ratio ∼ 2) [33]. By normalizing the RNA yields on fraction number 5, polysome profiles of different samples could be compared to each other. The addition of RNA yields from fractions 5 to 6 and 9 to 11 gave a hint on total RNA amount in gradients.

### Analysis of Amino Acid Metabolism

Free amino acids were analyzed by HPLC as described previously [34]. In brief, 10-fold diluted samples were derivatized using ortho-phthaldialdehyde/3-mercaptopropionic acid for primary and 9-fluorenylmethoxycarbonyl chloride for secondary amino acids [35]. Chromatographic separation was performed on a 250×4 Hyperclone ODS column protected by a precolumn (Phenomenex, Aschaffenburg, Germany). The gradient system consisted of 40 mM phosphate buffer (pH 7.8) and increasing amounts (6–100%) of a mixture containing acetonitril/methanol/water (45/45/10). Fluorimetric detection of primary and secondary amino acid derivatives was performed at 340/450 (excitation/emission) and 266/305 nm, respectively. Dilution curves from supplemented standard mixtures of amino acids (A9906 Sigma, Munich, Germany) were used in order to assign retention times and to obtain quantitative results.

### Data Analysis and Statistics

The statistical analysis was performed using the one-way analysis of variance from the JMP 8 software. The graphs were illustrated by using graph pad prism (Graph pad Software, La Jolla, USA). Weight data were analyzed by using the student’s t-test. The activation of the different signaling proteins is shown as relative activation (phospho-specific/total). Female samples were normalized for 11-week control mice. They were set to 100% and the results of the DU6P mice are shown as percentage. Some data are presented as percentage of control, which indicated the part of the same age Fzt:DU control. The significance level was set at p<0.05. Data are presented as mean ± SEM. The ratio protein/DNA was statistically evaluated with the Wilcoxon-signed rank test with the help of the SAS program.

## Results

### Success of Phenotype Selection and Weight Parameters

**Figure 1:**
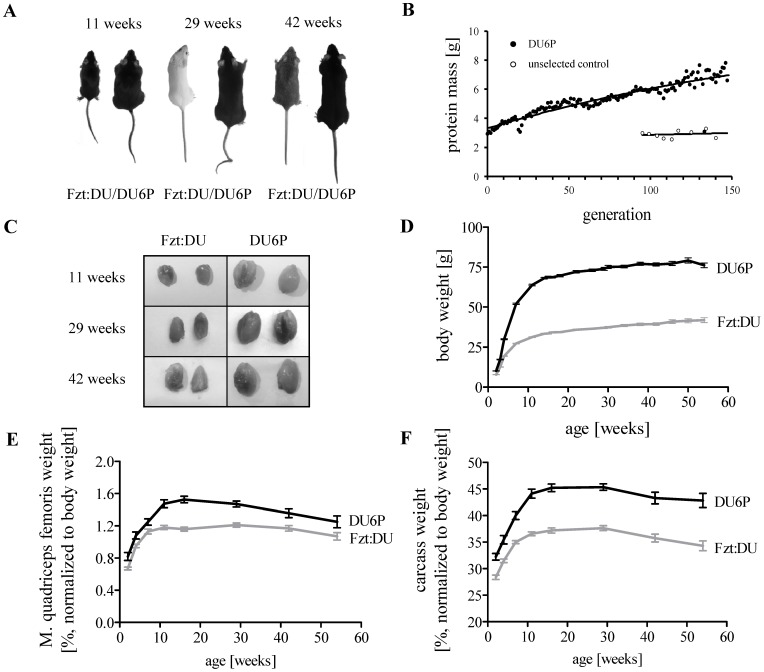
Long-term phenotype selection for high protein mass in mice. A: Phenotypes of female Fzt:DU and DU6P mice at the age of 11, 29 and 42 weeks. B: Success for the selected trait (protein mass) in the course of long-term selection in DU6P mice versus unselected control mice (Fzt:DU). C: Dissected *M. quadriceps femoris* of 11, 29 and 42-week female Fzt:DU and DU6P mice. D: Longitudinal body weights in female DU6P mice versus Fzt:DU mice. E and F: Relative weights of *M. quadriceps femoris* and carcasses from female Fzt:DU and DU6P mice (n = 15; p<0.03 for all comparisons of age-matched mouse lines). The sample number (n) depicts the number of samples per age group, the error bars represent SEM.

Long-term phenotype selection in DU6P mice for high protein mass resulted in a considerable selection success during the experiment. At the beginning of the selection period the protein content of 42 day old male mice amounted to 3.4 g. After 138 generations of selection for high protein mass the protein content increased to 7.8 g (p<0.001; Figure 1 A and B). Selection for high protein content also affected the absolute body mass in female DU6P mice if compared to the respective control mice in all age groups measured between week 2 and 54 after birth. Absolute weights of isolated muscles *(M. quadriceps femoris*, muscles are illustrated in Figure 1 C*)* in female DU6P mice were significantly higher (p<0.05) in all age groups studied if compared to Fzt:DU mice, respectively. Depending on age DU6P mice were characterized by up to 2-fold higher body weights compared to their controls (p<0.001; Figure 1 D). If corrected for body weights and calculated as relative muscle weights, muscle wet masses were higher in all age groups (Figure 1 E; p<0.03) demonstrating particular growth effects in muscles from DU6P mice in response to trait selection. Pronounced growth effects were not restricted to *M. quadriceps femoris* but seemed to be present also in other muscle tissues since the relative carcass weights from DU6P mice compared to controls were significantly increased if compared to controls (p<0.0001; Figure 1 F). Relative liver weights were similar, while relative brain weights were reduced in female DU6P mice and controls (p<0.001, data not shown). The effects of phenotype selection on weight parameters were similar in males and in females (data not shown).

### Serum Levels of IGF-I and IGF-II

**Figure 2:**
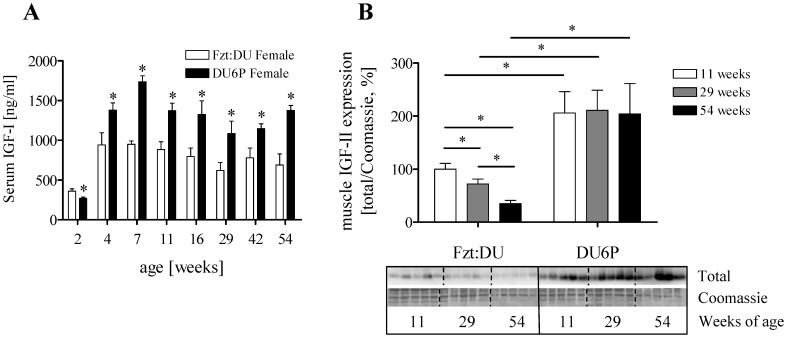
A: Serum IGF-I levels in female Fzt:DU and DU6P mice over life time (n = 8 per age group). B: Expression of IGF-II precursor (23 kDa) in muscle tissues from 11-, 29- and 54-week female Fzt:DU and DU6P mice (n = 10 per age group). The results are normalized for the signal intensities as detected by Coomassie blue staining of the membranes used for Western immuno detection. Data are expressed as percent of female 11-week Fzt:DU mice (100%). The error bars represent SEM.

Female DU6P mice had significantly higher IGF-I serum levels in all groups studied from 4 to 54 weeks of age if compared to Fzt:DU mice (p<0.05). In contrast, 2-week DU6P female mice exhibited lower IGF-I serum levels (p = 0.0153, Figure 2 A). Serum IGF-II levels were unaffected by age or phenotype in 11, 29 and 54-week female mice (not shown).

### Muscle Precursor IGF-II

In muscles from female DU6P mice the precursor of IGF-II was detected in significantly increased amounts compared to Fzt:DU (11 weeks: 206% of control, 29 weeks: 292% of control, 54 weeks: 585% of control; all p<0.0001; Figure 2 B).

### Analysis of Signal Transduction

**Figure 3:**
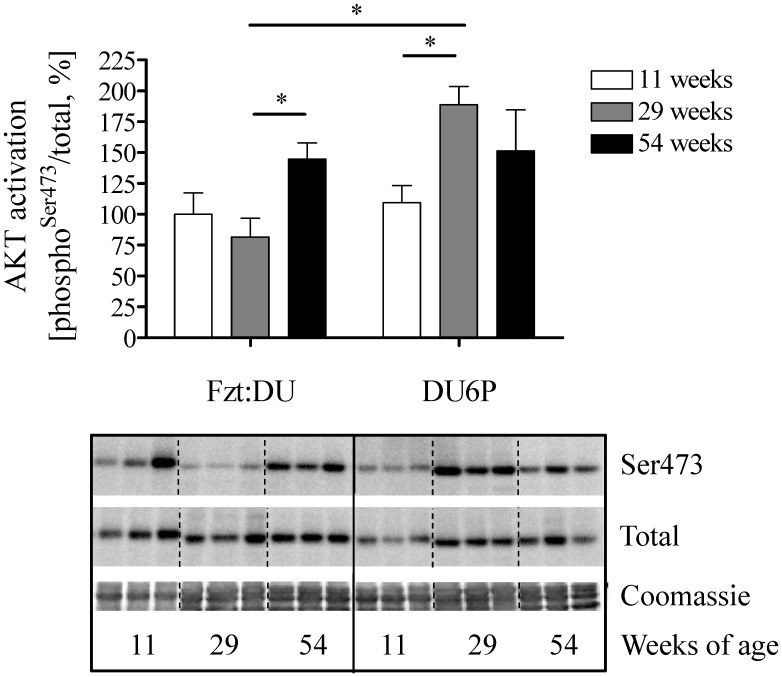
Analysis of signal transduction in muscle lysates from 11-, 29- and 54-week female DU6P and Fzt:DU mice. The Western blot inserts show phosphorylated (Ser-473) and total expression of AKT from one representative experiment, whereby all samples were studied on the identical membrane. Each Western blots were performed three times. Thus a total of 9 different animals was included in the bar chart for each timepoint. Specific activation was calculated from the ratios of phosphorylated versus total AKT (n = 9 per age group). Coomassie blue staining of the membranes used for Western immuno detection was used as loading control. The error bars represent SEM.

To get an overview on longitudinal activation of signaling pathways, we have performed an initial pool analysis (n = 15) in different age groups between 2 and 54 weeks and both sexes (2, 4, 7, 11, 16, 29. 42 and 54 weeks; data not shown). In the pool samples from female DU6P mice, activation of AKT on Ser-473 was strongly increased at an age of 16 (Fzt:DU: 54%; DU6P: 414%) and 29 weeks (Fzt:DU: 11%; DU6P: 510%). Although on a lower level, in males an increase was also detected in 16 (Fzt:DU: 75%; DU6P: 249%) and 29-week DU6P mice (Fzt:DU: 114%, DU6P: 162%). No consistent pattern of PDK1 activation could be identified in both DU6P groups if compared to Fzt:DU mice. The patterns of p44/42 and p38 MAPK activation were similar in DU6P and Fzt:DU mice in both sexes, respectively. If compared to females, male DU6P and Fzt:DU mice displayed higher levels of p38 or p44/42 MAPK activation particularly at the age of 29 weeks. As a result of our screening protocol for the activation of central signalling cascades in the longitudinal approach we considered activation of AKT in adult female mice as the most significant finding for further analysis. The expression and activation of AKT was studied also in unpooled samples (n = 9) from 11-, 29- and 54-week female mice of both lines. In fact, specific activation of AKT at Ser-473 was higher in 29-week mice if compared to 11-week mice (p = 0.0199) of the same phenotype and to 29-week control mice (p<0.0001; Figure 3). Activation of AKT at Thr-308 could not be detected in whole cell lysates but only as faint bands present in the isolated plasma membrane fraction (data not shown).

### Mechanisms of AKT Activation in Muscle

**Figure 4:**
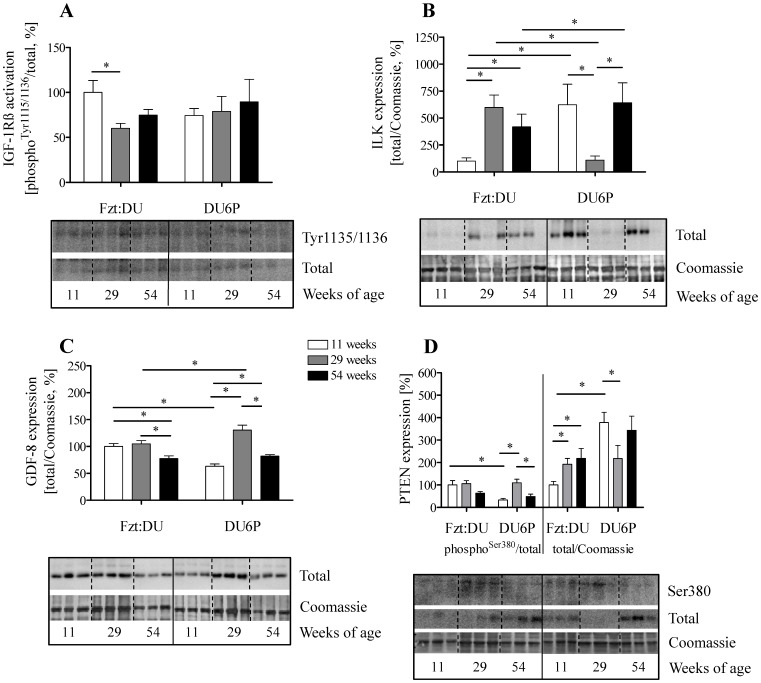
Analysis of signal transduction in muscle lysates from 11, 29 and 54-week female DU6P and Fzt:DU mice. Western blot identified phosphorylated and total expression of the respective signaling molecule. Specific activation was calculated from the ratios of phosphorylated versus total protein. Coomassie blue staining of the membranes used for Western immuno detection was used as loading control. A: IGF-1Rß in membrane fractions (n = 3); B: ILK (n = 9); C: GDF-8, 26 kDa band (n = 11) and D: PTEN (n = 9). The inserts provide representative experiments, whereby all samples were studied on the identical membrane. Sample numbers (n) depict the number of samples per age group, the error bars represent SEM.

In order to identify mechanisms responsible for altered activation at Ser-473 we have studied known effectors of AKT in female mice (Figure 4). IGF-1Rß expression and phosphorylation was detected in purified plasma membranes from muscle tissues (Figure 4 A). However no significant differences could be measured for the activation of IGF-1Rß in the different age and genetic groups studied from female mice. ILK expression was almost undetectable in tissue extracts of 29-week female DU6P mice (18% of control, p<0.0001; Figure 4 B) if compared to females of 11 and 54 weeks of age. In addition, ILK localization in the cytoplasmic membrane fraction was significantly reduced in 54-week female DU6P mice if compared to 11-week DU6P mice, while in control mice no difference was detectable (data not shown). Next, also expression of myostatin (GDF-8), as an important inhibitor of muscle accretion, was investigated. However, in 29-week female DU6P mice higher expression of the active 26 kDa peptide of myostatin was detected if compared to all other groups from the same phenotype and to the control mice, respectively (Figure 4 C).

Activation of AKT at Ser-473 is inhibited by phosphatase PTEN, which was strongly decreased in 29-week female DU6P mice (Figure 4 D). Although expression of total PTEN was severely reduced, we were able to identify increased levels of PTEN phosphorylation in 29-week female DU6P mice if compared to 11-week DU6P mice.

### Analysis of AKT Substrates

**Figure 5:**
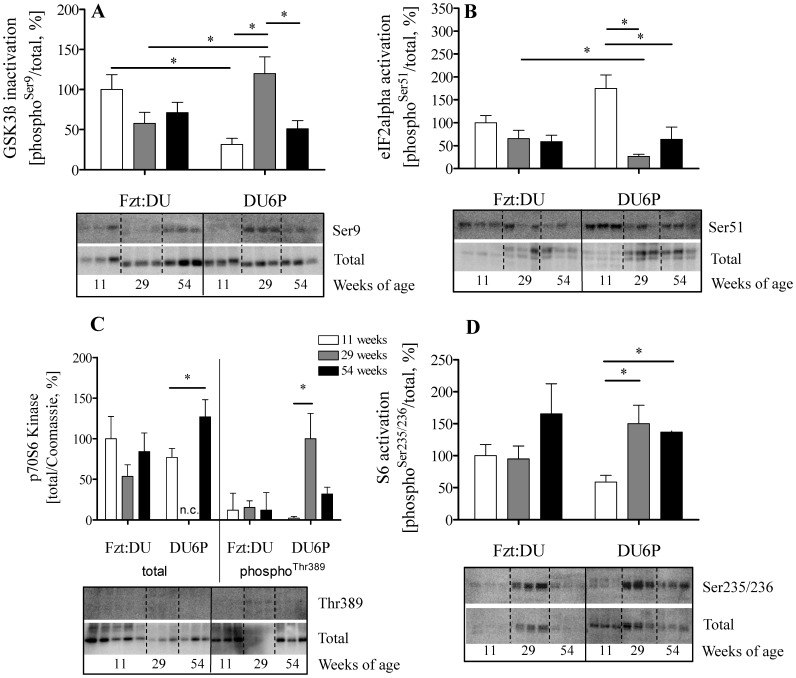
Analysis of signal transduction in muscle lysates from 11, 29 and 54-week female DU6P and Fzt:DU mice. Western blot identified phosphorylated and total expression of the respective signaling molecule. Specific activation was calculated from the ratios of phosphorylated versus total protein. A: GSK3ß (n = 9); B: eIF2α (n = 9); C: p70S6 kinase (n = 3); D: ribosomal protein S6 (n = 6). The inserts provide representative experiments, whereby all samples were studied on the identical membrane. Sample numbers (n) depict the number of samples per age group,the error bars represent SEM (n.c.: not calculated due to low signal intensity).

AKT controls growth and metabolism by phosphorylation of several enzymes (Figure 5). Phosphorylation of GSK3ß as a direct target of AKT was more than 2-fold (p<0.05) increased in 29-week female mice if compared to 11- and 54-week mice. In addition, reduced (p<0.05) levels of eIF2α phosphorylation in 29-week female DU6P mice were detected if compared to 11-week DU6P mice and 29-week control mice. Expression of total p70S6 kinase was down regulated in 29-week DU6P female mice when compared to all other age groups studied in DU6P and Fzt:DU (p<0.05). In spite of very low total p70S6 kinase expression, phosphorylated p70S6K could be detected in muscles from 29-week female DU6P mice. If normalized for the low expression levels specific activation of p70S6K was significantly higher if compared to 11- and 54-week female DU6P mice (p<0.05). We also studied activation of the S6 ribosomal protein. In 29-week female DU6P mice we identified significant activation if compared to 11-week female DU6P mice (p<0.05).

### Immunhistochemical and Biochemical Analysis of Glycogen in Muscle Tissue

**Figure 6:**
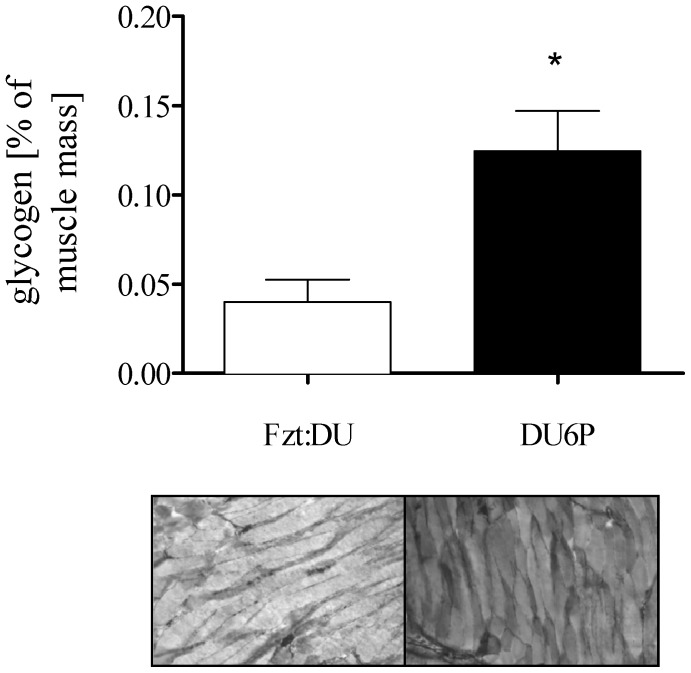
Higher levels of muscle glycogen in 29-week female DU6P mice. Top: Biochemical analysis of glycogen content in muscle tissue of 29-week female Fzt:DU (n = 6) and DU6P (n = 12) mice. Lower: PAS staining of cryosectioned muscle of 29-week Fzt:DU and DU6P female mice. The images correspond to an area of about 1.3×0.88 mm in the histological sections. The error bars represent SEM.

Robust inactivation of GSK3ß was correlated with 3-fold increased levels of muscle glycogen in 29-week female DU6P mice if compared to Fzt:DU (Fzt:DU: 0.04±0.01%; DU6P: 0.12±0.02%; p = 0.0079; Figure 6). This observation was confirmed by histochemistry.

### Protein Synthesis Versus Protein Breakdown

**Figure 7:**
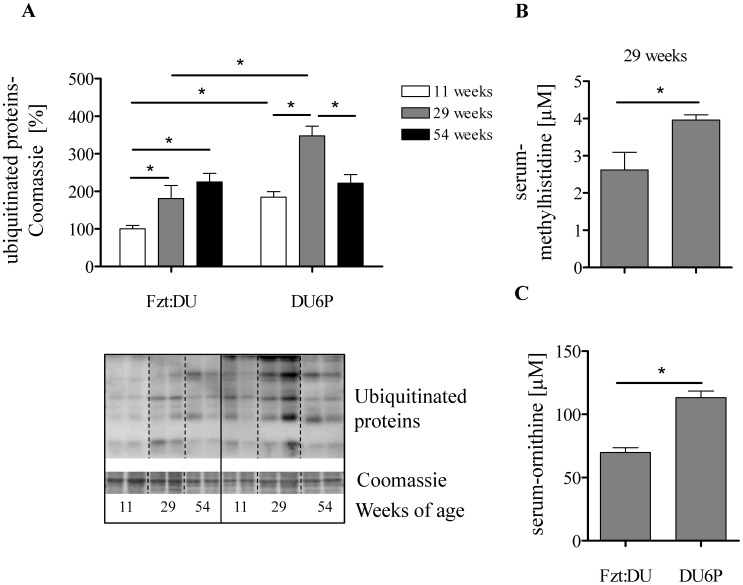
Analysis of protein metabolism in female DU6P and Fzt:DU mice. A: Western blot of muscle lysates in 11-, 29- and 54-week DU6P and Fzt:DU mice identified different proteins tagged with Ubiquitin for protein degradation (n = 5 per age group). Intensities of the complete lanes were calculated and normalized for the Coomassie blue signals present in the identical complete lane on the membranes used for Western immuno detection. The insert provides a representative experiment, whereby all samples were studied on the identical membrane. Serum levels of 1/3-methyl-histidine (B) and ornithine (C) were analyzed by quantitative HPLC as described in Materials and Methods. The error bars represent SEM.

The acquisition of muscle mass can further be achieved by higher protein synthesis and/or by a reduction of protein breakdown. We thus have analyzed activity of the ubiquitin/proteasome pathway by looking at global patterns of protein ubiquitination. In female 11- and 29-week DU6P mice higher levels of ubiquitinated proteins were detected when compared to age-matched Fzt:DU mice (p<0.05, Figure 7 A). In serum from female DU6P mice concentrations of 1/3-methyl-histidine as a marker of protein degradation and ornithine as a key molecule of the urea-cycle, were elevated if compared to controls (p<0.05, Figure 7 B and C). As a second marker of protein degradation also α-aminoadipic acid was significantly higher in 29-week DU6P mice compared to the controls (data not shown). While excluding reduced breakdown as a mechanism of muscle mass acquisition in DU6P mice we investigated the control of protein synthesis in DU6P mice and controls (table 2). Notably, female 29-week DU6P mice had a higher protein/DNA ratio when compared to age-matched controls as shown by the Wilcoxon-signed-rank test (Spearmen’s rank correlation coefficient is not significantly different from 0, i.e. both control groups are significantly different from 29-week DU6P mice). As RNA/DNA ratios were similar in all groups, we thus investigated if translational initiation was specifically affected and studied the relative distribution of mRNA present in polysomal versus non-polysomal fractions. While the relative distribution of free versus polysomally bound mRNA transcripts were similar in both genetic groups (table 2), not arguing for higher translation initiation, total amount of mRNA transcripts bound in polysomal complexes was significantly increased in 29-week female DU6P mice when compared to Fzt:DU mice (p<0.05).

**Table 2:** Analysis of body weight, contents of DNA, RNA and protein in *M. quadriceps femoris* of Fzt:DU (29 weeks) and DU6P (11 and 29 weeks) female mice (n = 5).

	DU6P 11 weeks	DU6P 29 weeks	Fzt:DU 29 weeks
body weight (g)	64.6±3.6^a^	70.9±1.1^a^	35.4±1.1^b^
muscle weight (*M. quadriceps femoris* (%)	1.5±0.1^a^	1.5±0.1^a^	1.2±0.1^b^
total DNA (µg)	521.3±56.5^a^	577.3±97.9^a^	299.6±48.9^b^
total RNA (µg)	305.1±31.0^a^	370.1±108.2^a^	139.9±45.8^b^
total Protein (mg)	378.1±46.7^a^	519.3±56.0^b^	149.9±5.1^c^
RNA/DNA (µg/µg)	0.6±0.1^a^	0.7±0.2^a^	0.6±0.3^a^
protein/DNA (mg/µg)	0.7±0.1	1.2±0.3*	0.6±0.1
protein/RNA (mg/µg)	1.3±0.2^a^	2.2±0.8^a^	1.6±0.4^a^
normalized non-polysomal RNA fraction (NP)	0.77±0.29^a^	0.73±0.33^a^	0.68±0.35^ a^
normalized polysomal RNA fraction (P)	0.58±0.28^a^	0.43±0.19^a^	0.64±0.47^a^
relative translational activity (P/NP)	0.63±0.07^a^	0.58±0,10^a^	0.64±0.40^a^
total translational activity (P/NP)*RNA	192±22^a^	213±38^a^	90±56^b^

a,b - different superscripts indicate significant differences (p<0.05);

* - significantly different if compared to 11-week DU6P or Fzt:DU, respectively as evaluated using the Wilcoxon-signed rank test.

Furthermore the non-polysomal and polysomal RNA fraction in *M. quadriceps femoris* of Fzt:DU (29 weeks) and DU6P (11 and 29 weeks) female mice (n = 4) was analysed.

**Figure 8:**
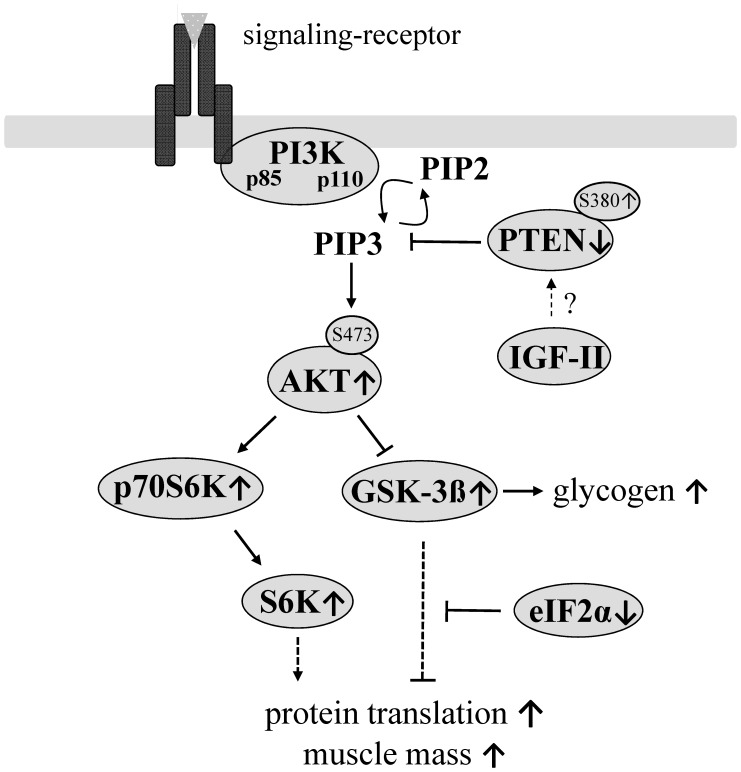
In mid-aged female DU6P mice inhibition of PTEN by means of reduced PTEN expression and increased PTEN phosphorylation correlates with higher levels of AKT phosphorylation at Ser-473, inactivation of GSK3ß on one hand and higher levels of S6K on the other. An increase of muscle mass is due to higher levels of muscle glycogen and an increased rate of protein synthesis in female 29-week DU6P mice.

## Discussion

### A New Mouse Model to Study Complex Control of Muscle Accretion

We have used a novel mouse model characterized by extreme muscle accretion and maintenance throughout lifetime. The mouse model has been developed by repeated phenotype selection for high protein content in 42 day old male mice. The selection experiment was performed for 138 generations, corresponding to a time span of more than 35 years and has been described in detail before [26], [36]. Since the selection experiment has been performed on an outbred background (60–80 breeding pairs per cycle) it can be assumed that a complex setting of alleles is contributing to the marked phenotype expressed by DU6P mice. Furthermore, we have to assume multiple mechanisms, positively affecting muscle accretion, have been emerged during the selection experiment. Our model represents an important tool of functional genome analysis and for estimating the physiological relevance of gene functions identified by transgenic or knockout models. An inherent feature of non-inbred models in part is extremely high phenotype variation. For selected parameters high phenotype variability was also found in the present study. As described in Materials and Methods, the phenotype of extreme protein accretion was established by crossbreeding 60–80 pairs in parallel during the complete selection experiment by keeping inbred coefficients as small as possible. Thus higher genetic complexity may be responsible for a higher phenotype variance also in DU6P mice. While high phenotype variability due to higher genetic complexity may complicate physiological studies, it basically can be considered also as a strength of the model, which represents not only a restricted genetic background. Thereby, phenotype based models add positive value to known gene functions by informing about their respective physiological relevance. Thus, our model is of central interest for general physiology, since it reveals strategies for the accretion and maintenance of muscle mass as invented and applied during evolution.

### Analysis of Signal Transduction from Weaning to Adulthood

To study mechanisms of muscle accretion and maintenance we monitored longitudinal activation of intracellular signaling cascades in female mice. According to current concepts AKT represents a central effector of muscle growth [37]. Therefore, we have screened pooled muscle lysates from 8 different age groups in the postnatal period between 2 and 54 weeks of age for the activation of AKT on Ser-473. Clearly, the profiles of AKT activation were different in mice selected for high protein accretion and randomly selected in mice. In adult female DU6P mice, extreme muscle mass coincided with massive increases of AKT activation in 16- and 29-week mice if compared to older or younger mice. The strong increase of AKT activation in 29-week female DU6P mice was confirmed on the level of single sample analysis. The reason for the absence of a stronger AKT activation, particularly in pubertal mice, characterized by higher growth rates, is unclear but was not expected. Ser-473 phosphorylation of AKT is thought to depend particularly on mTOR, present in complex 2 [38], [39]. Since mTOR phosphorylation was comparably low in 29-week female DU6P mice (Ser-2448), control of mTOR activity is not a plausible mechanism for the acute activation of AKT (data not shown). It has further been mentioned that full activation of AKT is dependent on Thr-308 phosphorylation by PDK1 [40]. However, the levels of phosphorylated AKT at Thr-308 were on very low levels suggesting that there is no robust impact of PDK1 via AKT in our model. In addition, activation of PDK1 itself was not affected in DU6P mice if compared to controls (data not shown). Abundance and phosphorylation of muscular IGF-I receptors were also found on very low levels arguing against acute regulation by growth factors via the IGF-I receptor/PDK1/AKT axis on muscle maintenance in adult female mice [41]. Although IGF-I serum levels were higher in adult female DU6P mice if compared to controls, acute regulation between 11- and 29-week female DU6P mice was absent and therefore not correlated with the acute activation of AKT. Interestingly, local IGF-II expression also ranged on higher levels in DU6P mice if compared to control mice. In pigs a QTL was mapped to the *IGF2* gene locus on chromosome 2 with a strong effect on lean mass in fact supporting functional relevance of IGF-II for muscle mass [42]. However, IGF-II expression in our mouse model cannot directly be used as a potential explanation for the acute activation of AKT in 29-week female mice, since the levels of serum IGF-II were similar in 11- and 29-week female DU6P mice. Nevertheless we cannot exclude conditional effects of local IGF-II in 29-week female mice particularly in a context of PTEN (see further down).

As one of the prominent inhibitors of muscle accretion, myostatin has been identified in mice [43], [44] and cattle [45], [46]. Notably, myostatin via TGFß-receptors and SMAD2/3 negatively acts on AKT phosphorylation [47]. However, myostatin expression was strongly increased in 29-week female DU6P mice, which did not explain the robust phenotype of protein accretion in adult female DU6P mice.

Recent evidence has been provided for the control of AKT activity by integrins [48]. Particularly, ILK has been demonstrated to have a strong effect on Ser-473 phosphorylation of AKT [49]. However, ILK was severely downregulated between week 11 and week 29 of age and thus almost undetectable in muscular tissue extracts from 29-week female DU6P mice. Since ILK is recruited to the plasma surface in the course of AKT activation, we have isolated a plasma membrane fraction from muscles of both genetic groups. In fact we were able to detect higher amounts of ILK bound to the cytoplasmic membrane if compared to whole tissue extracts, however again with reduced levels in 29-week female DU6P mice, which thus also was not explaining the increased levels of phosphorylated Ser-473-AKT.

As the exclusive motive for the strong increases of Ser-473-AKT phosphorylation, protein levels of PTEN were found to drop severely in 29-week female DU6P mice between 11 and 54 weeks of age. PTEN as a phosphatase catalyzes dephosphorylation of PIP3 to PIP2 and blocks activation of AKT [50], [51]. In spite of lower levels of total PTEN expression in muscles from 29-week female DU6P mice, we detected higher levels of phosphorylated PTEN, which is a known mechanism of PTEN inactivation [52], [53]. Although it is impossible to judge on the relative impacts of lower expression versus higher inactivation of PTEN, we have identified two independent mechanisms of PTEN-dependent evolved during the long term selection process for the control of AKT activity exerted on one hand, by down regulation of the PTEN protein amount and on the other, by inactivation of remnant PTEN. While Ser-380 of PTEN can be phosphorylated by casein kinase 2 [54] the control of PTEN expression [55] or degradation [56] is more complex. In the cytosol, PTEN expression represents a central regulatory mechanism for the control of AKT in malignant and non malignant conditions [57], [58]. Interestingly, the lack of PTEN in knockout mice [59] was sufficient to increase wet weight of the striated muscles (*M. tibialis anterior*). Further work is required in order to study the basis of altered PTEN expression or stability in female DU6P mice. In future studies also a potential role of local IGF-II for PTEN expression needs to be concerned since in human breast cancer cells a feedback loop comprising IGF-II and PTEN has been described by Perks et al. [60].

### Molecular Targets of Phosphorylated AKT in Female DU6P Mice

AKT represents a central control element for a plethora of effects on growth and metabolism [61], [62]. With respect to muscle accretion predominantly the AKT/p70S6 kinase/S6 kinase pathway is thought to be important [63], [64]. However, in muscles from 29-week female DU6P expression of p70S6 kinase surprisingly was almost undetectable and reduced if compared to 29-week female controls. In spite of severely reduced expression, phosphorylated p70S6K was found in 29-week female DU6P mice. In addition, robust and specific activation of S6 kinase was detected in 29-week female DU6P mice. Furthermore, an almost 2-fold specific activation of GSK3ß was measured in muscles from 29-week female DU6P mice. In an unphosphorylated form GSK3ß is known to phosphorylate and thereby inhibit glycogen synthase [65]. Phosphorylation of GSK3ß results in higher glycogen levels due to the lack of glycogen synthase inhibition. In fact, intramuscular glycogen levels were about 3-fold increased in 29-week female DU6P mice when compared to controls. It is important to note that muscle glycogen also may be due to altered muscle fiber composition, since glycolytic white muscle fibers contain more glycogen. However at least after 40 generations of selection fiber composition was similar in both strains [66].

In addition, the phosphorylation of eIF2α negatively regulates protein synthesis by inhibiting the ability of eIF2B to properly exchange GTP for GDP and is known to have a role in growth control [67], [68]. While the patterns of total eIF2 α where similar in both strains, reduced phosphorylation of eIF2α was exclusively found in 29-week female DU6P mice. Thus several mechanisms have evolved in female 29-week DU6P mice in response to growth selection: S6K, GSK3ß and eIF2α all have known positive effects on protein synthesis. Higher amounts of DNA in DU6P mice are in line with higher numbers of cell nuclei in individual muscle fibers (increases by up to 39%) and increases of fiber cross sections (about 55%) in DU6P mice as described by Rehfeldt and Bünger [66]. Notably, fiber numbers were identical in DU6P mice and controls. Interestingly, if normalized for higher DNA content as an estimate of active genomes, protein/DNA ratios were 2-fold increased in 29-week female DU6P mice if compared to Fzt:DU mice, in fact revealing higher rates of protein expression per cellular unit present in muscle fibers from 29-week female DU6P mice. We were able to demonstrate that DU6P mice have similar ratios of free mRNA versus mRNA bound to polysomes if compared to controls arguing against a higher initiation rate for protein translation. However, the amount of mRNA transcripts present in polysomes was much higher in 29-week female DU6P mice if compared to controls. On the other hand, we were able to exclude a reduction of protein breakdown as a mechanism of higher protein abundance in DU6P mice.

### Conclusions

As a summarizing scheme, Figure 8 depicts mechanisms identified in 29-week female DU6P mice. First our results clearly demonstrate that selection for high protein mass also selects for high muscle glycogen. This effect can unambiguously be related to the AKT/GSK3ß pathway. For the acute activation of this pathway exclusively inactivation of PTEN, by means of protein expression and protein phosphorylation, can be used in order to explain specific activation of AKT at Ser-473 in 29-week female DU6P mice. We furthermore emphasize that the control of muscle mass seems to occur in an age-dependent fashion since PTEN is affected specifically in mid-aged but not in pubertal or advanced aged female mice. In addition, we were able to argue against acute involvement of a number of known effectors of AKT including serum IGF-I and IGF-II, IGF-I receptor activation, PDK1-, mTOR-, myostatin- and ILK-expression in 29-week female DU6P mice. Since the mechanism presented is established by a total of 138 rounds of phenotype selections, we have reason to include a particular physiological relevance to PTEN levels if the accretion and maintenance of muscle tissue in mid-aged female mice is concerned.
